# Serotonin re-uptake transporter gene polymorphisms are associated with imatinib-induced diarrhoea in chronic myeloid leukaemia patients

**DOI:** 10.1038/s41598-020-65350-0

**Published:** 2020-05-21

**Authors:** Andrea Davies, Ana Eugenia Rodriguez-Vicente, Gemma Austin, Sandra Loaiza, Letizia Foroni, Richard E Clark, Munir Pirmohamed

**Affiliations:** 1 The Wolfson Centre for Personalised Medicine, The MRC Centre for Drug Safety Science, Molecular and Clinical Pharmacology, University of Liverpool, Liverpool, UK; 2 Molecular and Clinical Cancer Medicine, University of Liverpool, Liverpool, UK; 3 Department of Haematology, Hammersmith Hospital, Imperial College London, London, UK; 4 Present Address: IBMCC-Cancer Research Center, University of Salamanca, and Hematology Department, Hospital Universitario Salamanca, Salamanca, Spain

**Keywords:** Predictive markers, Gastrointestinal hormones, Adverse effects

## Abstract

Tyrosine kinase inhibitors (TKIs), the treatment of choice for chronic myeloid leukaemia (CML), can cause lower gastrointestinal (GI) toxicity which is manifested as diarrhoea. The mechanisms are not fully understood. The enteroendocrine signalling compound, serotonin (5-HT), is important for regulating peristaltic motion, fluid secretion and visceral hypersensitivity in the GI tract, and has been implicated in diseases such as irritable bowel syndrome. In this study, we have evaluated whether TKI-induced diarrhoea may be related to variation in the serotonin re-uptake transporter (SERT) gene. CML patients with and without diarrhoea on the SPIRIT2 trial (imatinib, n = 319; and dasatinib, n = 297) were genotyped for the promoter 5-HTTLPR, intron 2 VNTR and rs25531 polymorphisms by PCR-based methods. Diarrhoea was more prevalent in imatinib, than in dasatinib treated patients (P = 0.015), which when stratified by gender was seen to be driven by female patients (P = 0.036). Logistic regression analysis revealed that age, and the dominant HTTLPR with the rs25531 single nucleotide polymorphism (SNP) model, explained the occurrence of diarrhoea in ~10% of imatinib-treated female CML patients. These data suggest SERT polymorphisms influence imatinib-induced diarrhoea but not that of dasatinib.

## Introduction

Tyrosine kinase inhibitor drugs (TKIs) are now the mainstay of treatment for chronic myeloid leukaemia (CML). Several are now available, and a meta-analysis of first-line treatment options for CML showed that there was little difference seen across all TKIs regarding complete cytogenetic response (CCyR), progression-free survival (PFS) and overall survival (OS) outcomes^1^. The choice of therapy can be dictated by many factors including the occurrence of adverse events (AEs).

Diarrhoea is a common socially debilitating AE associated with TKIs. Imatinib causes diarrhoea of any grade in ~25% of CML patients^2^. Higher imatinib dose, in the phase 2 SWOG S0325 trial, increased the incidence, but not necessarily the grade of diarrhoea from 39% to 56%^3^. Dasatinib caused diarrhoea in 1 in 5 patients in the DASISION trial, similar to that seen with imatinib^4^. By contrast, the second generation TKI, bosutinib, was responsible for diarrhoea in up to 84% of patients in the BELA trial^5^ and the NCT00261846 phase1/2 study^6^. The mechanisms or predisposing factors for TKI-related diarrhoea are poorly understood.

In broad terms, diarrhoea can occur through either secretory or enteroendocrine/neuronal pathways. Secretory diarrhoea results from ion flux across the cell membrane that potentiates the movement of water, a phenomenon called ‘solvent drag’. Enteroendocrine induced diarrhoea is stimulated by neurotransmitters such as serotonin (5-hydroxytryptamine; 5-HT), vasoactive intestinal peptide and acetylcholine which can alter motility and fluid secretion of the gut^7^.

Serotonin (5-HT) is important for regulating peristaltic motion, fluid secretion and visceral hypersensitivity in the gastro-intestinal (GI) tract^7^. Approximately 90% of the body’s total 5-HT is located within enterochromaffin cells (ECs) in the GI epithelium. Release of 5-HT at neuronal synapses and into the circulation stimulates bowel urgency and movement by binding to 5-HT receptors that predominate in the GI tract^8^. Regulation of circulatory levels of 5-HT is performed by the serotonin reuptake transporter (SERT). Both GI epithelial cells and platelets express high levels of SERT and are therefore capable of sequestering excess 5-HT which, when required, can be released as part of a tissue damage response^7^.

The SERT protein is encoded by the SLC6A4 (solute carrier family 6 member 4) gene, which has a highly polymorphic promoter region – the serotonin-transporter-linked polymorphic region (5-HTTLPR) (rs4795541)^9^. A common insertion/deletion (indel) polymorphism within the 5-HTTLPR generates two transcripts, a long allele (L) containing a sequence of 43 or 44 base pairs (depending on the presence of a single deletion single nucleotide polymorphism (SNP) rs1462091560), and a short allele (S) in which this sequence is deleted^9^. The short form is associated with 2–3-fold reduced SERT expression when compared to the long form^10–12^. The rs25531 SNP G-allele, located within the 5-HTTLPR, creates a functional AP2 transcription-factor binding site which converts the L-allele phenotype to that of the S-allele^13^. The intronic STin2 VNTR 17 bp tandem repeat (rs57098334) has 4 known alleles consisting of 12, 10, 9 and 7 repeats. The functional influence of the 12 and 10 tandem repeats were tested in a transgenic embryonic mouse model which showed that the longer sequence acted as a stronger transcriptional inducer than the shorter sequence^14^.

Circulating levels of 5-HT are elevated in patients who suffer from diarrhoea-related irritable bowel syndrome (IBS-D) while those who experience the constipation equivalent (IBS-C) disease have low 5-HT levels^15^. The association of polymorphic variants within the 5-HTTLPR promoter region with IBS has been inconsistent^16–19^, with a recent meta-analysis of 27 IBS studies in Asian and Caucasian individuals showing that 5-HTTLPR polymorphisms did not correlate with IBS-D^20^. However, the SERT knock-out mouse also experiences increased bowel motility and diarrhoea^21^. Interestingly, bosutinib, which has the highest incidence of diarrhoea amongst the TKIs, inhibits SERT by 71% as an off-target effect^22^ suggesting bosutinib-induced diarrhoea may occur through elevated circulating 5-HT levels. We therefore hypothesised that TKI-induced diarrhoea is a result of increased circulating 5-HT levels with genetic polymorphisms in the SERT gene acting as predisposing factors. In order to investigate this, we have evaluated the commonly reported intronic STin2 VNTR, and the promoter (5-HTTLPR) ‘long’ and ‘short’ indel variants with the rs25531 SNP, in imatinib and dasatinib treated CML patients as part of the SPIRIT2 randomised trial.

## Results

### Patient characteristics

Of the patients recruited to the SPIRIT2 trial, we excluded patients from this study for the following reasons: 1) no consent or sample given; 2) mention of constipation; 3) co-morbidities involving the GI tract; 4) documented co-medications that would influence diarrhoea or serotonin levels including selective serotonin re-uptake inhibitors; and 5) no PCR product due to poor DNA. This left 319 imatinib and 297 dasatinib-treated patients who were finally included in the study (Supplementary Fig. 1). There were no significant differences between imatinib and dasatinib patient groups regarding age, BMI or platelet count (Table 1).

**Table 1:** Patient clinical information stratified by drug and gender.

Characteristics	imatinib			dasatinib			Comparison between TKI		
	No. (%) of patients			No. (%) of patients			P value (95% CI)		
	total	male	female	total	male	female	total	male	female
*Gender*	319	186 (58.3)	133 (41.7)	297	188 (63.3)	109 (36.7)	0.206 (−0.127, 0.027)		
Age at diagnosis (years)									
mean	54	54	53	53	52	54	0.404 (−1.353, 3.356)	0.097 (−0.463, 5.603)	0.429 (−5.270, 2.248)
range	20–87	22–87	20–83	18–89	18–89	20–88			
<65	243 (76.2)	139 (74.7)	104 (78.2)	227 (76.4)	151 (80.3)	76 (69.7)	0.941 (−0.065, 0.070)	0.193 (−0.029, 0.141)	0.134 (−1.958, 0.026)
≥ 65	76 (23.8)	47 (25.3)	29 (21.8)	70 (23.6)	37 (19.7)	33 (30.3)			
*BMI*									
mean	27.9	26.8	27.8	27.0	27.2	26.8	0.056 (−0.021, 1.750)	0.142 (−0.266, 1.852)	0.202 (−0.554, 2.609)
range	19.0–59.8	19.0–59.8	19.8–46.6	18.5–44.2	18.5–43.9	19.3–44.2			
Missing data	20 (6.3)	12 (6.5)	8 (6.0)	20 (6.7)	8 (4.3)	12 (11.0)			
*Platelet count (x10*^9^*/L)*									
mean	513	463	582	501	444	598	0.688 (−46.875, 71.010)	0.585 (−49.086, 86.829)	0.762 (−120.722, 88.492)
range	16–2714	57–2212	16–2714	77–3140	77–2433	107–3140			
Missing data	3 (0.9)	3 (1.6)	0 (0)	2 (0.7)	2 (1.1)	0 (0.0)			
*Diarrhoea incidence*									
Yes	144 (35.7)	55 (29.6)	59 (44.4)	79 (26.6)	45 (23.9)	34 (31.2)	**0.015 (0.018, 0.165)**	0.219 (−0.034, 0.146)	**0.036 (0.009, 0.255)**
No	205 (64.3)	131 (70.4)	74 (55.6)	218 (73.4)	143 (76.1)	75 (68.8)			
*Toxicity grade*									
1	86 (75.4)	42 (76.3)	44 (74.5)	56 (70.9)	35 (77.8)	21 (61.8)	0.120 (−0.020, 0.173)	0.231 (−0.044, 0.182)	0.408 (−0.100, 0.246)
2	16 (14.0)	9 (16.4)	7 (11.9)	15 (19.0)	5 (11.1)	10 (29.4)			
3	1 (0.9)	0 (0.0)	1 (1.7)	2 (2.5)	1 (2.2)	1 (2.9)			
4	0 (0.0)	0 (0.0)	0 (0.0)	0 (0.0)	0 (0.0)	0 (0.0)			
Missing data	11 (9.7)	4 (7.3)	7 (11.9)	6 (7.6)	4 (8.9)	2 (5.9)			

A T-test of independent samples was performed to compare differences between drug groups. Text in bold indicates statistical significance (P < 0.05). BMI = body mass index. Prevalence of diarrhoea is classified as incidence (yes or no) and toxicity grade (1 to 4).

### Allelic frequencies

The rs25531 SNP and 5-HTTLPR promoter indel polymorphism (rs4795541) did not deviate from the Hardy-Weinberg Equilibrium (HWE) within the imatinib and dasatinib-treated CML cohorts (Table 2). The tri-allelic STin2 VNTR polymorphism however showed significant deviation from HWE in both imatinib (χ^2^ = 24.1537; P = 0.0002) and dasatinib (χ^2^ = 19.0302; P = 0.0019) arms. Our observed minor allele frequency (MAF) for rs25531 was 7%, which is consistent with the reported Caucasian frequency of 9% on the HaploReg v4.1 website^23^. A study performed in 2622 European-American subjects, using the National Institute of Mental Health repository, found that the 5-HTTLPR short allele frequency was 43%^24^, which is identical to our findings. The frequencies of the STin2 VNTR 12, 10 and 9 alleles in our cohort were 62%, ~36% and ~2.5% respectively. Our frequency of 12 and 10 alleles are consistent with various addiction and disorder studies within a predominantly Caucasian background, but we have detected twice the frequency of 9 allele carriers than these published studies^25–28^. Our major deviation from the HWE was also replicated by these groups. We did not identify any carriers of the 7-repeat length STin2 VNTR in our patient cohort.

**Table 2:** Hardy-Weinberg Equilibrium (HWE) calculations of patient SERT genotypes.

Genotype		all patients (n = 616)					imatinib patients (n = 319)					dasatinib patients (n = 297)				
		obs (n)	exp (n)	allele freq.	χ^2^	P value	obs (n)	exp (n)	allele freq.	χ^2^	P value	obs (n)	exp (n)	allele freq.	χ^2^	P value
5-HTTLPR (rs4795541)	L	194	198				99	103				95	95			
	LS	311	302	L = 0.5674			164	156	L = 0.5674			147	146	L = 0.5673		
	S	111	115	S = 0.4326	0.4972	0.780	56	60	S = 0.4326	0.7119	0.701	55	56	S = 0.4327	0.0199	0.990
rs25531	A	535	535				277	278				258	259			
	AG	78	78	A = 0.9318			41	40	A = 0.9326			37	37	A = 0.9341		
	G	3	3	G = 0.0682	0.0075	0.996	1	1	G = 0.0674	0.1600	0.923	2	1	G = 0.0659	0.0723	0.964
STin2 VNTR (rs57098334)	12/12	242	236				121	122				121	113			
	12/10	262	272				145	142				117	130			
	12/09	16	19				8	9				8	10			
	10/10	86	79	12 = 0.6185			41	41	12 = 0.6191			45	38	12 = 0.6178		
	10/09	6	11	10 = 0.3571			2	5	10 = 0.3589			4	6	10 = 0.3552		
	09/09	4	<1	09 = 0.0244	39.8473	**0.00001**	2	<1	09 = 0.0219	24.1537	**0.0002**	2	<1	09 = 0.0269	19.0302	**0.0019**

P value is a measure of the deviation from HWE. Text in bold indicates significant P values. Serotonin-transporter-linked polymorphic region (5-HTTLPR). Intron 2, 17 bp variable number of tandem repeats (STin2 VNTR); 12, 10 and 9 tandem repeat alleles (12, 10 and 09 respectively). Allele frequency (allele freq.). Observed genotype (obs); expected genotype (exp); “long” allele (L); “short” allele (S). Text in bold indicates statistical significance (P < 0.05).

### Incidence of diarrhoea is more prevalent with imatinib treatment and driven by gender and 5-HTTLPR genotype

**Figure 1:**
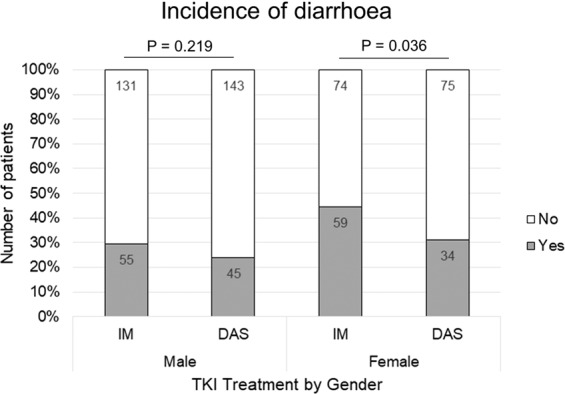
Diarrhoea incidence (yes or no) experienced by TKI treated CML patients. Patients stratified by imatinib (IM) and dasatinib (DAS) treatment and gender. Histogram represent data from Table 1. Actual patient numbers are incorporated within the histogram bars.

A comparison of means test demonstrated the imatinib group had a greater incidence of diarrhoea compared with dasatinib (P = 0.015; 95% CI = 0.018, 0.165). When separated by gender, this significance was seen to be driven by female (P = 0.036; 95% CI = 0.009, 0.255) but not male patients (P = 0.219; 95% CI = −0.034, 0.146) (Table 1 & Fig. 1A).

Logistic regression analysis incorporating all patients (n = 616) showed drug (P = 0.015), gender (P = 0.002), age (P = 0.049), BMI (P = 0.034) and the dominant HTTLPR model (P = 0.049) were significant covariates for the dependent variable of diarrhoea (Table 3). After retaining these covariates in a forward conditional regression analysis, only gender and BMI were retained in the model explaining diarrhoea as an adverse event in 3.4% of the patient cohort.

**Table 3:** Logistic regression analysis of incidence of diarrhoea, by drug treatment, in CML patients.

Covariate		all patients			imatinib patients			dasatinib patients		
		r^2^	P value	OR (95% CI)	r^2^	P value	OR (95% CI)	r^2^	P value	OR (95% CI)
Drug		0.014	**0.015**	0.652 (0.462, 0.920)	—	—	—	—	—	—
Gender		0.021	**0.002**	0.585 (0.414, 0.826)	0.031	**0.007**	0.527 (0.331, 0.838)	0.009	0.174	0.694 (0.410, 1.174)
Age		0.009	**0.049**	1.012 (1.000, 1.024)	0.004	0.344	1.008 (0.992, 1.024)	0.016	0.077	1.016 (0.998, 1.033)
Age (≥ 65)		0.004	0.202	1.291 (0.872, 1.910)	0.003	0.436	1.234 (0.726, 2.098)	0.005	0.297	1.367 (0.760, 2.459)
BMI		0.011	**0.034**	1.035 (1.003, 1.069)	0.009	0.158	1.031 (0.988, 1.075)	0.009	0.176	1.035 (0.985, 1.088)
Platelets		0.000	0.997	1.000 (1.000, 1.000)	0.000	0.997	1.000 (0.999, 1.001)	0.000	0.955	1.000 (0.999, 1.001)
5-HTTLPR	biallelic	0.003	0.258	0.867 (0.676, 1.111)	0.023	**0.021**	0.666 (0.472, 0.940)	0.004	0.383	1.178 (0.815, 1.702)
	rec	0.000	0.782	0.940 (0.605, 1.459)	0.000	0.997	1.001 (0.548, 1.828)	0.001	0.643	0.858 (0.448, 1.641)
	dom	0.009	**0.049**	0.696 (0.486, 0.999)	0.052	**0.0004**	0.417 (0.255, 0.680)	0.004	0.358	1.304 (0.740, 2.299)
rs25531	biallelic	0.002	0.371	1.235 (0.777, 1.963)	0.001	0.587	1.195 (0.629, 2.270)	0.003	0.451	1.296 (0.660, 2.545)
5-HTTLPR + rs25531	biallelic	0.002	0.400	0.900 (0.704, 1.150)	0.024	**0.020**	0.663 (0.469, 0.937)	0.008	0.200	1.267 (0.882, 1.821)
	rec	0.002	0.409	1.187 (0.790, 1.783)	0.007	0.221	1.429 (0.807, 2.528)	0.000	0.813	0.932 (0.519, 1.673)
	dom	0.001	0.587	0.897 (0.606, 1.328)	0.027	**0.012**	0.509 (0.301, 0.862)	0.018	0.064	1.845 (0.965, 3.528)
STin2 VNTR	triallelic	0.002	0.366	1.074 (0.920, 1.255)	0.017	0.043	1.252 (1.007, 1.557)	0.003	0.470	0.917 (0.725, 1.160)
	model B	0.002	0.364	1.100 (0.896, 1.351)	0.016	0.057	1.323 (0.992, 1.764)	0.002	0.513	0.902 (0.663, 1.228)
	model A	0.001	0.477	1.092 (0.857, 1.390)	0.008	0.162	1.271 (0.908, 1.778)	0.001	0.666	0.924 (0.646, 1.322)
	model A rec	0.002	0.348	0.803 (0.508, 1.270)	0.022	**0.022**	0.476 (0.252, 0.899)	0.004	0.373	1.389 (0.674, 2.864)
	model A dom	0.000	0.746	1.059 (0.747, 1.503)	0.000	0.765	1.075 (0.670, 1.725)	0.000	0.961	1.013 (0.600, 1.712)
Covariate retained in model		r^2^			r^2^					
Gender		0.023			—					
Gender with BMI		0.034			—					
5-HTTLPR dom		—			0.052					
5-HTTLPR dom with gender		—			0.078					

Body mass index (BMI). Age, in years, as a continuous variable (Age). Age, in years, defined as under 65, or 65 and over, as a binary variable (Age ≥ 65). Serotonin-transporter-linked polymorphic region alone (5-HTTLPR); or combined with rs25531 SNP (5-HTTLPR + rs25531). Intron 2, 17 bp variable number of tandem repeats (STin2 VNTR); allelic model definitions described in Supplementary Table 2. Recessive allele (rec); dominant allele (dom). Text in bold indicates covariates included in the forward conditional regression model.

On further separation of the patients by the drug received, however, none of the tested covariates were significant for the dasatinib cohort. The significant differences were driven by the imatinib-treated patient cohort, and included gender (P = 0.007), 5-HTTLPR genotype (P = 0.020 and P = 0.021), especially of the dominant allele model (P = 0.012 and P = 0.0004), with or without the rs25531 SNP combined, respectively, and the STin2 VNTR (P = 0.043) polymorphism. After including these covariates in a forward conditional regression analysis, gender enhanced the 5-HTTLPR dominant model to explain diarrhoea in 7.8% of the imatinib-treated patients. By further stratifying the imatinib cohort by gender, only the 5-HTTLPR dominant model explained ~6% of male diarrhoea cases, but age and the 5-HTTLPR plus the rs25531 dominant model explained nearly 10% of diarrhoea cases for females (Table 4).

**Table 4:** Logistic regression analysis of incidence of diarrhoea, stratified by gender, in imatinib treated CML patients. Body mass index (BMI).

Covariate		male imatinib patients			female imatinib patients		
		r^2^	P value	OR (95% CI)	r^2^	P value	OR (95% CI)
Age		0.002	0.589	0.994 (0.973, 1.016)	0.055	**0.022**	1.032 (1.005, 1.060)
Age (≥ 65)		0.001	0.740	0.883 (0.424, 1.841)	0.030	0.084	2.091 (0.906, 4.827)
BMI		0.024	0.080	1.055 (0.994, 1.120)	0.000	0.926	1.003 (0.945, 1.064)
Platelets		0.007	0.355	0.999 (0.998, 1.001)	0.000	0.832	1.000 (0.999, 1.001)
5-HTTLPR	biallelic	0.029	**0.053**	0.610 (0.370, 1.007)	0.018	0.183	0.723 (0.448, 1.166)
	rec	0.001	0.799	0.892 (0.369, 2.156)	0.000	0.992	0.996 (0.425, 2.331)
	dom	0.049	**0.010**	2.420 (1.234, 4.744)	0.042	**0.042**	0.470 (0.227, 0.972)
rs25531	biallelic	0.010	0.245	1.638 (0.713, 3.766)	0.003	0.616	0.771 (0.279, 2.130)
5-HTTLPR + rs25531	biallelic	0.022	0.089	0.407 (0.407, 1.066)	0.028	0.099	0.652 (0.392, 1.084)
	rec	0.010	0.271	0.631 (0.278, 1.433)	0.005	0.470	1.353 (0.595, 3.075)
	dom	0.020	0.105	1.807 (0.884, 3.694)	0.038	**0.053**	0.449 (0.200, 1.009)
STin2 VNTR	triallelic	0.028	**0.054**	1.323 (0.996, 1.758)	0.009	0.338	1.185 (0.837, 1.678)
	model B	0.034	**0.035**	1.510 (1.030, 2.212)	0.003	0.561	1.143 (0.729, 1.792)
	model A	0.021	0.099	1.479 (0.929, 2.354)	0.001	0.761	1.081 (0.656, 1.781)
	model A rec	0.017	0.129	1.933 (0.825, 4.532)	0.031	0.081	2.444 (0.895, 6.669)
	model A dom	0.012	0.221	0.658 (0.337, 1.287)	0.007	0.394	1.357 (0.672, 2.742)
Covariate retained in model		r^2^			r^2^		
5-HTTLPR dom		0.059			—		
Age		—			0.055		
Age with 5-HTTLPR + rs25531 dom		—			0.097		

Age, in years, as a continuous variable (Age). Age, in years, defined as under 65, or 65 and over, as a binary variable (Age ≥ 65). Serotonin-transporter-linked polymorphic region alone (5-HTTLPR); or combined with rs25531 SNP (5-HTTLPR + rs25531). Intron 2, 17 bp variable number of tandem repeats (STin2 VNTR); allelic model definitions described in Supplementary Table 2. Recessive allele (rec); dominant allele (dom). Text in bold indicates covariates included in the forward conditional regression model.

**Figure 2:**
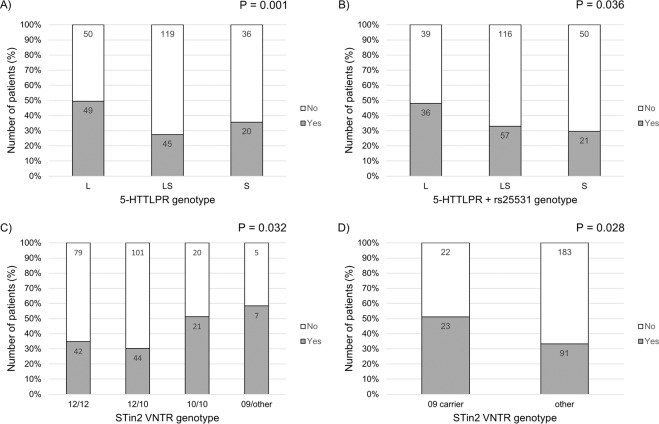
Chi-square analyses of SERT genotype and occurrence of diarrhoea (yes or no) in imatinib-treated patients. (**A**) 5-HTTLPR indel alone; (**B**) 5-HTTLPR indel with rs25531 SNP combined; (**C**) STin2 VNTR model B; and (**D**) STin2 VNTR model A. Genotype model codes are described in Supplementary Table 2. Actual patient numbers are incorporated within the histogram bars.

To ascertain which alleles influenced imatinib-induced diarrhoea incidence, we performed Chi-square analyses. The ‘long’ allele of the 5-HTTLPR genotype alone correlated with a greater incidence of diarrhoea (P = 0.001) (Fig. 2A). The association was still significant when 5-HTTLPR was combined with the rs25531 SNP, and displayed a modest gene dose-dependent trend (P = 0.036) (Fig. 2B) with the dominant ‘long’ allele driving this significance for the incidence of diarrhoea (P = 0.013) (Supplementary Table 3). When separated by gender, significant trends were not favoured by any one gender for the 5-HTTLPR dominant genotype (male: P = 0.012; female: P = 0.045) (Supplementary Table 3). Significance was lost when considering the 5-HTTLPR with rs25531 SNP combined, but a trend was observed favouring females with the dominant genotype (P = 0.066).

In patients with imatinib-induced diarrhoea, carriers of the 09 alleles for the STin2 VNTR were more likely to experience diarrhoea: model B (P = 0.032) (Fig. 2C) and recessive model A (P = 0.028) (Fig. 2D). When stratifying by gender this trend appeared to be influenced by females, though not significantly (Supplementary Table 3). However, these data must be viewed with caution owing to the low patient numbers of 09 allele carriers available for analysis: 9/186 (4.8%) male and 3/133 female (2.3%).

### SERT genotype does not correlate with diarrhoea grade

Diarrhoea toxicity grade was not significantly different between drug arms (P = 0.120; 95% CI = −0.020, 0.173) (Table 1). Diarrhoea experienced by all TKI-treated patients were largely grade 1 and 2 (75% and 14% of patients for imatinib, and 71% and 19% for dasatinib, respectively) (Table 1). Only three patients had grade 3 diarrhoea and there were no reports of grade 4 diarrhoea. Diarrhoea toxicity grade was not significantly different between drug arms or gender (Table 1). Chi-square analysis with genotype did not show any significant correlations with diarrhoea toxicity grade for either the imatinib or dasatinib arms (Supplementary Table 3).

## Discussion

Our analysis shows that in the SPIRIT2 trial, the incidence of diarrhoea was greater in imatinib- than in dasatinib-treated chronic myeloid leukaemia (CML) patients. Imatinib-induced diarrhoea was influenced by gender and SERT genotype. Carriers of the 5-HTTLPR ‘long’ allele or the STin2 VNTR 9-repeat allele were more likely to experience diarrhoea. Logistic regression identified that the dominant ‘long’ allele of the 5-HTTLPR explained ~6% of diarrhoea incidence in men, while age and the dominant ‘long’ allele of the 5-HTTLPR plus rs25531 SNP explained 10% of cases of diarrhoea in females. Furthermore, females younger than 65 were less likely to experience diarrhoea than those over the age of 65 (Supplementary Fig. 2). Although this trend was not significant, it is consistent with the findings with bosutinib where 92% over the age of 65 years experienced diarrhoea^6^. By contrast, dasatinib-induced diarrhoea was not correlated with any covariate tested.

To our knowledge this is the first study to investigate the role of SERT genotype in drug-induced diarrhoea. Our patients largely had grade 1 or 2 diarrhoea and we had no patients with grade 4 diarrhoea. However, grade 1 or 2 diarrhoeas (up to 1500 ml stool per day) can significantly impact a patient’s quality of life. The SERT genotype has been investigated in the diarrhoea predominant irritable bowel syndrome (IBS-D), but studies have been contradictory in their assessment of which 5HTTLPR allele, ‘long’ or ‘short’, is responsible for enhancing diarrhoea symptoms^29^. It is important to note that not all studies considered age or gender, or the rs25531 SNP that lies within the 5-HTTLPR region. Thus, many patients may have been incorrectly genotyped as ‘long’ when a G variant (L_G_) would in fact have converted this to a ‘short’ allele phenotype. Our analyses demonstrated some loss of significance with the incorporation of the rs25531 SNP within the 5-HTTLPR genotype highlighting the subtle difference this may cause in changing phenotype and reported outcomes. Furthermore, a modest allele-dose-dependent pattern, with diarrhoea incidence, was observed with the incorporation of the rs25531 SNP, compared with the 5-HTTLPR indel alone, suggesting that combining the 5-HTTLPR indel and rs25531 SNP displays a more accurate dose dependent phenotype.

Less emphasis has been placed on the STin2 VNTR polymorphism with fewer published studies investigating this polymorphism compared with 5-HTTLPR. The shorter 10-repeat was shown to reduce SERT expression compared to the full length 12-repeat allele^14^. The shorter 9-repeat was not investigated, but it may be inferred by analogy that this allele would cause further reduced expression of SERT^30^. Owing to the low frequency of the 09 allele, previous studies may have been under-powered (~100 patients) to determine any association^19^. Our cohort, however, comprised ~300 patients per drug treatment which allowed us to show a correlation between carriers of the 09 allele and imatinib-induced diarrhoea. With reduced expression of SERT being associated with a shorter STin2 VNTR sequence, our data support the hypothesis that a reduction in SERT activity would result in elevated circulating 5-HT concentrations leading to diarrhoea.

The 5-HTTLPR and rs25531 polymorphisms within our CML patients conformed to HWE. The deviation of the STin2 VNTR polymorphism from HWE may be related to its complex nature; we identified patients with 12, 10 and 9 alleles in our cohort of 616 patients but did not identify an individual with the 7-repeat allele, and hence our sample size may not have been sufficient to determine the true population allele frequency of a polymorphism which consists of four, or possibly more, yet to be identified, alleles. Furthermore, we did not identify any significant differences in patient demographics, such as age, gender, BMI and platelet count, between the imatinib and dasatinib arms, and therefore any differences seen in diarrhoea AEs between the TKI arms may be attributed to the effect of the drug.

IBS-D is more prevalent in women than in men^31^, and the 5-HT_3_ receptor antagonist alosetron is more effective against IBS-D in women than men^32^. It is therefore of interest that we found trends suggesting that the effect of the SERT genotype may be more important in females with imatinib-induced diarrhoea. Our study however would have benefited from the inclusion of more females to support, or refute, these trends into statistically significant findings.

We and other groups have only tested the three most commonly reported polymorphisms despite there being over 9700 documented SNPs within the SLC6A4/SERT gene^33^. Our *a priori* decision in not including further SNPs was due to the vast number and lack of functional information regarding all of these SNPs. Of course, the serotonin transporter is just one component in the serotonin pathway, which includes multiple 5-HT receptors (5-HTRs), the enzyme tryptophan hydroxylase 1 (TPH1) that is required to synthesise the supply of serotonin, as well as genes involved in post-receptor signalling cascades. Investigation of these SNPs may be worthwhile in the future, but will require a much larger sample size, and should probably proceed via a genome-wide association approach.

Our investigation was predicated on the hypothesis that TKI-induced diarrhoea was mediated through increased circulating 5-HT levels caused by SERT genotypes responsible for reducing SERT expression or activity. However, the associations we observed: (a) the ‘long’ allele 5-HTTLPR genotype, with or without rs25531, would reduce circulating 5-HT level, which is counterintuitive to our hypothesis; (b) carriers of the STin2 VNTR 09 allele, which reduces SERT expression, were more likely to experience diarrhoea, which does fit our hypothesis. It is clear that SERT functional activity is dependent on a number of factors, genetic and non-genetic, and is therefore highly complex. It is also possible that circulating 5HT levels may not necessarily correlate with 5HT concentrations in tissues, making the relationship with genotype even more complex. Nevertheless, the reason for undertaking this study does have a strong biological plausibility, and the findings of the study suggest that further investigations of the role of 5HT in the pathogenesis of diarrhoea caused by TKIs, which is particularly troublesome for patients, is worthwhile. It may also allow the use of 5HT modulating drugs in the treatment of patients so that they can continue their therapy for CML.

An interesting finding from our study is that the association was seen with diarrhoea induced by imatinib but not by dasatinib. Although dasatinib and imatinib are both classed as Bcr-abl inhibitors to treat CML, they vary with respect to the off-target tyrosine kinases that they inhibit^34^, and it is therefore possible that kinases within the bowel may be differentially affected by these drugs as off-target effects. An interaction between the 5HT system and the kinase may therefore be required to elicit diarrhoea. Since the mechanisms of TKI-induced diarrhoea have not been fully elucidated, this is an area which merits further investigation.

In conclusion, our study has identified an association between diarrhoea induced by imatinib, but not by dasatinib, and SERT polymorphisms. The strength of our study is that the patients were recruited from a randomised controlled trial (SPIRIT2) where patients will have had standardised assessment of efficacious and adverse events, as well as being randomised into different arms. However, despite the relatively large sample size, further studies will be needed to replicate this finding. It will also be important to understand the mechanistic basis of the role of 5HT in causing diarrhoea induced by TKIs, which should also include assessment of the off-target effects of the TKIs on the bowel kinome and the interaction with the 5HT pathway.

## Methods

### Patient recruitment

The SPIRIT2 trial is a phase 3 prospective randomised comparison of imatinib (400 mg) versus dasatinib (100 mg) each daily in newly-diagnosed chronic phase chronic myeloid leukaemia (CML) patients (www.ClinicalTrials.gov; study identifier: NCT01460693). Ethical permission for the trial was granted by the London Research Ethics Committee, UK (REC ref# 07/H0718/90). Written informed consent was obtained from patients prior to their inclusion and the study conducted according to the declaration of Helsinki. The principal results from the study have been presented previously^35^. The patients’ ethnicity was not catalogued, but they were assumed largely to be Caucasians as they were recruited within the UK. Each arm of the study recruited 407 patients with the primary outcome measure described as 5-year event free survival. GI adverse events (AEs) were logged as occurrence of diarrhoea (i.e. yes or no) and grade was classified according to the Common Terminology Criteria for Adverse Events (CTCAE) system^36^.

### DNA Extraction

Genomic DNA was extracted from diagnostic patient bio-banked mononuclear cells using the AllPrep DNA/RNA Mini Kit (Qiagen^®^, Manchester, UK). Quantification, and quality assessment, of the DNA, was performed on the Nanodrop 2000 UV-VIS Spectrophotometer (ThermoFisher Scientific, Loughborough, Leicestershire, UK). Samples were stored at −20 °C until required.

### SERT/SLC6A4 Genotyping

The SERT/SLC6A4 polymorphisms multiplex PCR assay was adapted from Wendland *et al*.^37^ Primers were synthesised by MWG Eurofins, Germany (Supplementary Table 1). Patient genomic DNA (80 ng) was amplified using OneTaq enzyme (Promega, Southampton, UK) with GC Buffer (New England Biolabs, Herts, UK), dNTPs (10 mM final conc.), and equimolar concentrations (50pmol final concentration) of forward and reverse primers for both 5-HTTLPR and VNTR. PCR cycling conditions were: 94 °C for 30 secs; 35 cycles of 94 °C for 30 secs; 64 °C for 30 secs; 68 °C for 30 secs; and 68 °C for 5 mins. To determine presence of the rs25531 SNP within the 5-HTTLPR region, a restriction enzyme digest was performed on the PCR product. Seven microliters of product was digested with MspI in CutSmart buffer (New England Biolabs, Herts, UK) at 37 °C for 90 mins. PCR products were separated by 2.5% 0.5x TBE-buffered agarose gel and visualised with ethidium bromide using a UV trans-illuminator.

Interpretation of the PCR products expected is described in Supplementary Table 1. Ten percent of the samples were duplicated, for assay reliability, and genotypes were reproducible and in complete concordance. SERT genotypes, dominant and recessive models, were classified as shown in Supplementary Table 2.

### Statistical analyses

Differences between treatment arms for age, gender, body mass index (BMI), platelet count and diarrhoea incidence were performed using a comparison of means test for independent samples. Correlations between genotype and categorical and ordinal outcomes were performed by Chi-square (χ^2^) analyses, generating Pearson and Fisher’s Exact tests. One-way ANOVA tests were performed with linear covariates. Logistic regression analyses were performed to describe the influence of covariates on diarrhoea. These analyses were performed using the IBM SPSS statistics software v24 (IBM Corp., Armonk, NY, USA).

## Supplementary information

Supplementary data.

## Data Availability

All data generated or analysed during this study are included in this published article (and its Supplementary Information files).
