# Supplementary material for: Serotonin re-uptake transporter gene polymorphisms are associated with imatinib-induced diarrhoea in chronic myeloid leukaemia patients
Source: Sci Rep. 2020 May 21;10:8394. doi: 10.1038/s41598-020-65350-0 (PMC7242433; doi:10.1038/s41598-020-65350-0)
Supplement: Supplementary file 1 — Supplementary data. [file 41598_2020_65350_MOESM1_ESM.pdf]

Serotonin re-uptake transporter gene polymorphisms are associated with imatinib-induced diarrhoea in chronic myeloid leukaemia patients.

Authors: Andrea Davies, Ana Eugenia Rodriguez-Vicente, Gemma Austin, Sandra Loaiza, Letizia Foroni, Richard E Clark, Munir Pirmohamed

Supplementary Data

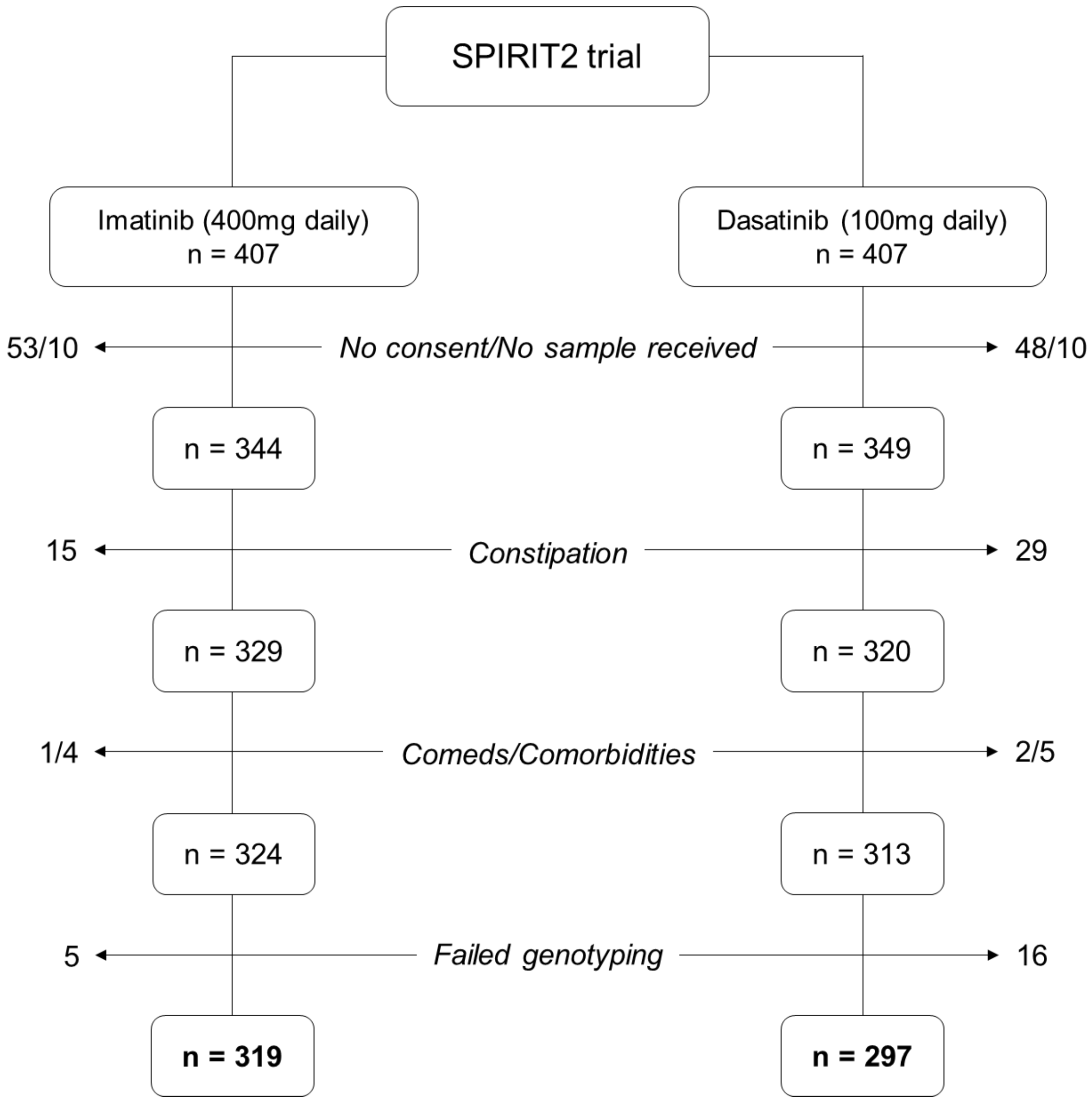

Supplementary Figure 1: Flow diagram of the number of chronic myeloid leukaemia (CML) patients, in both imatinib and dasatinib arms of the SPIRIT2 study, that were included for SERT genotyping. Patient receiving comedications known to cause diarrhoea (comeds).

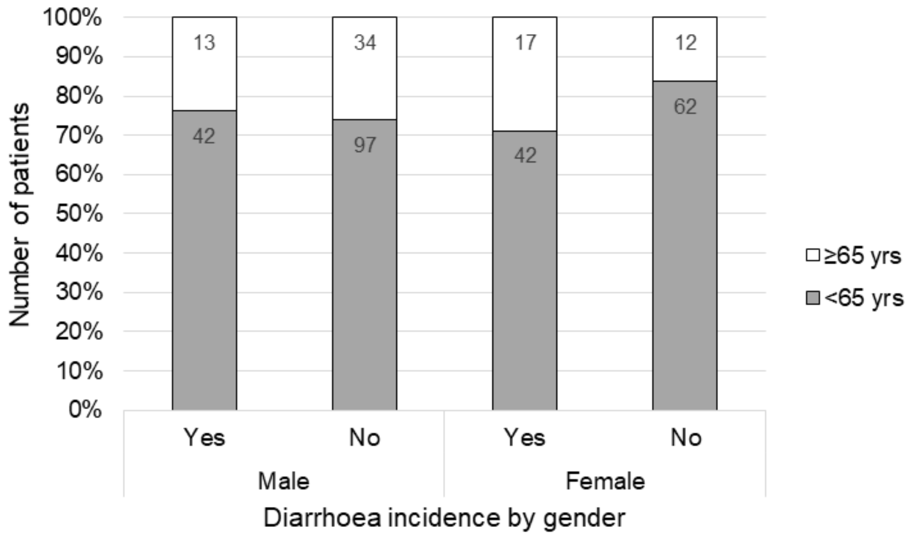

Supplementary Figure 2: Chi-square analyses of diarrhoea incidence (yes or no) by age and gender in imatinib-treated patients. Patients were binary classified as under 65 (<65 yrs) or 65 and above (≥65 yrs). Actual patient numbers are incorporated within the histogram bars.

**Primer sequences**	**PCR product**	**MspI digest**
5-HTTLPR & rs25531		
FWD: 5’-TCC TCC GCT TTG GCG CCT CTT CC-3’	S - 469bp	S_A - 469bp (uncut)
REV: 5’-TGG GGG TTG CAG GGG AGA TCC TG-3’		S_G - 402bp + 67bp
	L - 512bp	L_A - 512bp (uncut)
		L_G - 402bp + 110bp
STin2 VNTR		
FWD: 5’-GGG CAA TGT CTG GCG CTT CCC CTA CAT A-3’	STin2.09 - 248bp	-
REV: 5’-TTC TGG CCT CTC AAG AGG ACC TAC AGC-3’	STin2.10 - 265bp	-
	STin2.12 - 299bp	-

Supplementary Table 1: Forward (FWD) and reverse (REV) primer sequences for end point multiplex PCR of the human SERT/SLC6A4 gene. Serotonin-transporter-linked polymorphic region (5-HTTLPR); “long” allele (L) and “short” allele (S). Intron 2 variable number of tandem repeat (STin2 VNTR). MspI enzyme digestion of the PCR products indicates the presence of the rs25531 SNP variant G allele (S_G and L_G); undigested PCR product indicates the rs25531 reference A allele (S_A and L_A). Size of PCR product indicated by base pairs (bp).

SNP description		Corresponding Genotypes
5-HTTLPR (rs4795541)	biallelic	L v LS v S
	rec	S v (LS+L)
	dom	L v (LS+S)
rs25531	biallelic	A v AG v G
5-HTTLPR + rs25531	biallelic	L_AL_A v (L_AL_G + L_AS_A + L_AS_G) v (S_AS_A + S_GS_A + S_GS_G + L_GL_G + L_GS_A + L_GS_G)
	rec	(S_AS_A + S_GS_A + S_GS_G + L_GL_G + L_GS_A + L_GS_G) v other
	dom	L_AL_A v other
STin2 VNTR (rs57098334)	triallelic	12/12 v 12/10 v 12/09 v 10/10 v 10/09 v 09/09
	model B	12/12 v 12/10 v 10/10 v 09/other
	model A	12/12 v (12/10 + 12/09) v (10/10 + 09/10 + 09/09)
	model A rec	09 carriers v other
	model A dom	12/12 v other

Supplementary Table 2. Classification of SERT genotypes. Serotonin-transporter-linked polymorphic region (5-HTTLPR); “long” allele (L), “short” allele (S). Intron 2, 17bp variable number of tandem repeats (STin2 VNTR). Recessive allele (rec); dominant allele (dom).

		P value					
		Incidence				Toxicity Grade	
		imatinib			dasatinib	imatinib	dasatinib
Genotype		all	male	female			
5-HTTLPR (rs4795541)	biallelic	**0.001**	**0.030**	0.087	0.645	0.579	0.876
	rec	1.000	1.000	1.000	0.617	0.731	1.000
	dom	**0.001**	**0.012**	**0.045**	0.400	0.602	1.000
rs25531	biallelic	0.401	0.248	0.799	0.671	0.176	0.822
5-HTTLPR + rs25531	biallelic	**0.036**	0.211	0.145	0.158	0.724	0.390
	rec	0.262	0.330	0.539	0.881	0.730	0.368
	dom	**0.013**	0.127	0.066	0.071	0.566	0.720
STin2 VNTR (rs57098334)	triallelic	0.108	0.370	0.081	0.504	0.603	0.744
	model B	**0.032**	0.152	0.081	0.603	0.304	0.582
	model A	0.061	0.226	0.066	0.615	0.419	0.535
	model A rec	**0.028**	0.163	0.086	0.486	0.301	0.672
	model A dom	0.810	0.220	0.473	1.000	0.586	0.779

Supplementary Table 3. Chi-square analyses of SERT genotype with drug-treated diarrhoea incidence and toxicity grade (1 to 3). Serotonin-transporter-linked polymorphic region (5-HTTLPR); “long” allele (L) and “short” allele (S). Intron 2, 17bp variable number of tandem repeats (STin2 VNTR). Recessive allele (rec); dominant allele (dom). Genotype model codes are described in supplementary table 2. Significant p values in bold type.
